# Factors influencing hospital stay duration for patients with mild ischemic colitis: a retrospective study

**DOI:** 10.1186/s40001-022-00665-4

**Published:** 2022-03-05

**Authors:** Haosu Huang, Hanyue Wang, Zhenpu Long, Meng Wang, Junjie Ding, Jie Peng

**Affiliations:** Department of Gastroenterology, Xiangya Hospital, Central South University, 87 Xiangya Road, Changsha, 410008 Hunan China

**Keywords:** Ischemic colitis, Mild, In-hospital stay, Risk factors

## Abstract

**Background:**

Ischemic colitis is the most prevalent ischemic injury of the gastrointestinal tract. The majority of patients with mild ischemic colitis usually achieve complete clinical recovery shortly. However, the predictors of longer hospital stay duration are unclear. This study aimed to evaluate the predictors of hospital stay duration for patients with mild ischemic colitis.

**Methods:**

We retrospectively evaluated 100 patients with mild ischemic colitis between January 2010 and December 2020 at Xiangya Hospital (a tertiary care center). The clinical characteristics and therapeutic drugs of patients who were hospitalized for ≤ 8 days and ≥ 12 days were compared.

**Results:**

Of the 100 patients included, 63 (63%) were hospitalized for ≤ 8 days and 37 (37%) were hospitalized for ≥ 12 days. Patients with cerebrovascular disease (29.7% vs. 11.1%, *p* = 0.019) and abdominal surgical history (29.7% vs. 7.9%, *p* = 0.004) were more likely to be hospitalized for ≥ 12 days than for ≤ 8 days. The d-dimer levels [0.78 (0.41–1.82) vs. 0.28 (0.16–0.73), *p* = 0.001] and positive fecal occult blood test results (86.5% vs. 60.3%, *p* = 0.006) were higher in patients who were hospitalized for ≥ 12 days than in those who were hospitalized for ≤ 8 days. Probiotic use was greater in patients hospitalized for ≤ 8 days (76.2% vs. 54.1%, *p* = 0.022). Multivariate analysis indicated that cerebrovascular disease (odds ratio [OR] = 4.585; 95% confidence interval [CI] 1.129–18.624; *p* = 0.033), abdominal surgical history (OR = 4.551; 95% CI 1.060–19.546; *p* = 0.042), higher d-dimer levels (OR = 1.928; 95% CI 1.024–3.632; *p* = 0.042), and higher positive fecal occult blood test results (OR = 7.211; 95% CI 1.929–26.953; *p* = 0.003) were associated with longer hospital stays.

**Conclusion:**

Cerebrovascular disease, abdominal surgical history, higher d-dimer levels, and higher positive fecal occult blood test results are independent and significant factors that influence longer hospital stays for patients with mild ischemic colitis. Probiotics helped reduce hospital stay in these patients.

## Background

Ischemic colitis (IC) is defined as the inflammation of the colon secondary to vascular insufficiency and ischemia, with abdominal pain, hematochezia, and diarrhea being the most common manifestations [1]. IC is the most common form of intestinal ischemia, with an annual incidence of 15.6–17.7 per 100,000 individuals [2]. The severity of ischemic necrosis varies from superficial injury to the mucosa and submucosa to full-thickness transmural necrosis of the colonic wall [3]. The incidence of IC has continued to increase [4], and it often occurs in older patients, patients with comorbidities, and women. Additionally, IC is an increasingly common cause of hospitalizations related to lower gastrointestinal bleeding [5].

More than three-fourths of IC cases are the milder, nongangrenous form; however, IC can progress to necrosis with a risk of mortality up to 50% [6]. Treatment would depend on the severity at presentation. Most IC cases are benign, transient, and self-limiting, and symptoms typically resolve within 2–3 days, with the colon completely healing within 1–2 weeks [7]. Mild IC usually benefits from conservative management and supportive care [8].

To date, multiple studies have reported numerous risk factors for IC, including cerebrovascular disease, hypertension, diabetes mellitus, previous history of abdominal surgery, irritable bowel syndrome, and constipation [9]. However, no published studies have addressed the significant predictors of longer hospital stay duration for patients with mild IC. Therefore, the purpose of this study was to identify factors that are associated with longer hospital stays for these patients. We attempted to identify patients with mild IC who were more likely to be hospitalized for > 12 days and the drugs that contribute to shortened hospital stays and improved future management.

## Patients and methods

### Patients

This retrospective study included a total of 100 patients who were diagnosed with mild IC and admitted to Xiangya Hospital (a comprehensive tertiary care center) between January 2010 and December 2020. A search of the Xiangya Hospital electronic medical records system was performed, and 166 diagnoses of IC were recorded during the evaluation period. The final study population comprised 100 patients with mild IC. The following variables were analyzed: clinical variables, including age, sex, clinical manifestations, past medical history, and chronic medication for mild IC; laboratory variables, including d-dimer level, erythrocyte sedimentation rate, and fecal occult blood test (FOBT) results; computed tomography (CT) findings typical of IC; and endoscopic variables. Since most IC cases are self-limiting, and symptoms typically resolve within 2–3 days, with the colon completely healing within 1–2 weeks [7], the length of hospital stay of patients with mild IC was approximately 7–14 days. Thus, the patients were classified into those hospitalized for ≤ 8 days and those hospitalized for ≥ 12 days. Clinical examination results, drug treatment, laboratory findings, and imaging and endoscopic data were collected from the medical records documented at the time of diagnosis.

All the procedures performed in this study were conducted in accordance with the ethical standards of the institutional research committee and the 1964 Helsinki Declaration and its later amendments or comparable ethical standards. The requirement for obtaining informed consent was waived because of the retrospective nature of the study, and the study protocol was approved by the Ethics Committee of Xiangya Hospital, Central South University (No. 202012711).

It was not appropriate or possible to involve patients or the public in the design, or conduct, or reporting, or dissemination plans of our research.

### Diagnosis and inclusion criteria

The diagnosis of IC was confirmed based on a typical medical history, CT, colonoscopy, and histological examination of the colonoscopy specimen. The distribution of the disease was determined according to the following colonoscopy results: left-sided, disease distributed from the splenic flexure to the rectum; right-sided, disease distributed from the cecum to the distal transverse colon; and bilateral, disease involving both sides of the colon. Disease severity was classified as mild, moderate, or severe according to the criteria proposed in the American College of Gastroenterology (ACG) guidelines [8]. Mild disease was considered a segmental colitis, which is not isolated to the right colon, and had no risk factors associated with poor outcomes observed with moderate disease. The criterion for discharge was the absence of clinical symptoms, with or without secondary colonoscopy results, which indicated that the intestinal mucosa had returned to normal.

In total, 123 patients with complete medical records who were diagnosed with mild IC over an 11-year period were initially identified. Of these, 100 patients who were hospitalized for ≤ 8 days or ≥ 12 days were included in this study. Patients who were diagnosed with IC for the first time and hospitalized because of it were eligible for inclusion in this study. In contrast, the exclusion criteria included a diagnosis of inflammatory bowel disease, recent antibiotic use, and positive fecal sampling for enteric infections. Patients with prolonged hospitalizations because of other diseases or complications and those who were discharged in advance because of economic challenges were also excluded.

### Statistical analysis

Statistical analyses were performed using SPSS version 26.0 software (SPSS, Inc., Chicago, IL, USA). The continuous variables are expressed as means ± standard deviations or medians and interquartile ranges, as appropriate. The categorical variables are expressed as frequencies (*n*, %). Normal distributions were ascertained using the Shapiro–Wilk test before parametric tests were performed. The categorical variables were analyzed using a Chi-square test, and the continuous variables were analyzed using the Mann–Whitney *U* and Student’s *t*-tests, as appropriate. A logistic regression analysis was performed to identify significant predictors associated with longer hospital stays for patients with mild IC, including model variables with a *p*-value < 0.05 in the univariate analysis. Odds ratios (ORs) are presented with 95% confidence intervals (CIs). A *p*-value < 0.05 was considered statistically significant.

## Results

### Patient characteristics

Table 1 shows the baseline characteristics of the patients. Among the 100 patients with mild IC who were included in the study, 63 and 37 patients were hospitalized for ≤ 8 days and ≥ 12 days, respectively. The mean length of hospital stay was 9.6 days. The baseline characteristics, including age, sex, and clinical manifestations, were similar for both groups. The study population had a mean age of 61.50 ± 11.31 years, and 62% of the patients were women. The most common symptom was abdominal pain, which was experienced by 86 (86%) patients. Other symptoms included rectal bleeding (76 patients; 76%), decreased appetite (28 patients; 28%), and diarrhea (16 patients; 16%). Only 16 (16%) patients exhibited nausea and/or vomiting and 19 (19%) had abdominal distension. Overall, 49% of the patients had hypertension, 19% had a history of coronary heart disease, 18% had cerebrovascular disease, 21% had diabetes mellitus, and 16% had a history of abdominal surgery. There were significantly more patients with a history of cerebrovascular disease in the group of patients that were hospitalized for ≥ 12 days than in the group of patients hospitalized for ≤ 8 days (29.7% vs. 11.1%, *p* = 0.019). Patients with a history of abdominal surgery (29.7% vs. 7.9%, *p* = 0.004) were more likely to be hospitalized for ≥ 12 days than those without.

**Table 1:** Comparison of the baseline characteristics and hospital stay duration for patients with mild IC

Variable	Total	Hospital stay duration ≤ 8 days	Hospital stay duration ≥ 12 days	*p*-value
Patients	100	63	37	–
Age, years	61.50 ± 11.31	61.22 ± 11.72	61.97 ± 10.70	0.75
Sex				
Male	38	25 (39.7)	13 (35.1)	0.65
Female	62	38 (60.3)	24 (64.9)	
Clinical manifestations				
Abdominal pain	86	55 (87.3)	31 (83.8)	0.63
Rectal bleeding	76	48 (76.2)	28 (75.7)	0.95
Diarrhea	16	11 (17.5)	5 (13.5)	0.6
Nausea and/or vomiting	16	9 (14.3)	7 (18.9)	0.54
Decreased appetite	28	17 (27.0)	11 (29.7)	0.77
Abdominal distension	19	11 (17.5)	8 (21.6)	0.61
Fever	1	1 (1.6)	0	0.44
Weight loss	9	7 (11.1)	2 (5.4)	0.34
Previous medical history				
Diabetes mellitus	21	17 (27.0)	4 (10.8)	0.055
Atherosclerosis	9	8 (12.7)	1 (2.7)	0.092
Hyperlipidemia	20	16 (25.4)	4 (10.8)	0.078
Hypertension	49	29 (46.0)	20 (54.1)	0.44
Coronary heart disease	19	9 (14.3)	10 (27.0)	0.12
Cerebrovascular disease	18	7 (11.1)	11 (29.7)	0.019
Fatty liver disease	11	7 (11.1)	4 (10.8)	0.96
Atrial fibrillation	2	1 (1.6)	1 (2.7)	0.7
Cholecystitis	16	11 (17.5)	5 (13.5)	0.6
History of abdominal surgery	16	5 (7.9)	11 (29.7)	0.004

### Clinical examination results

Table 2 shows the results of the clinical examinations. All the included patients underwent a colonoscopy. The most common findings were erythema (77%) and bowel edema (76%). Nearly all the patients who underwent a colonoscopy had mild endoscopic findings, without necrosis or deep ulceration. A superficial colonic ulcer was identified in 11% of these patients. Considering both the endoscopic and CT findings, lesions were most frequently observed in the left colon (distal to the splenic flexure) (*n* = 67, 67%), and bilateral colonic involvement was observed in only 33% of the cases. None of the patients exhibited isolated involvement of the right colon. No significant difference in the number of lesions was observed between the two groups. The laboratory results showed that the d-dimer levels were significantly higher in patients who were hospitalized for ≥ 12 days than in those hospitalized for ≤ 8 days [0.78 (0.41–1.82) vs. 0.28 (0.16–0.73), *p* = 0.001]. Positive FOBT results were also higher in the patients who were hospitalized for ≥ 12 days than in those hospitalized for ≤ 8 days (86.5% vs. 60.3%, *p* = 0.006).

**Table 2:** Comparison of examination results and hospital stay duration for patients with mild IC

Variable	Total	Hospital stay duration ≤ 8 days	Hospital stay duration ≥ 12 days	*p*-value
Patients	100	63	37	–
Colonoscopy findings				
Erythema	77	46 (73.0)	31 (83.8)	0.22
Edema	76	47 (74.6)	29 (78.4)	0.67
Multiple punctate erosions	29	15 (23.8)	14 (37.8)	0.14
Superficial ulcer	11	4 (6.3)	7 (18.9)	0.05
Distribution				
Left-sided	67	44 (69.8)	23 (62.2)	0.43
Right-sided	0	0	0	0
Bilateral	33	19 (30.2)	14 (37.8)	0.43
Number of lesions	2 (1–3)	2 (1–3)	2 (1–6)	0.066
Computed tomography findings				
Bowel wall thickening and/or edema	14	12 (19.0)	2 (5.4)	0.06
d-dimer (mg/L)	0.47 (0.18–1.31)	0.28 (0.16–0.73)	0.78 (0.41–1.82)	0.001
Erythrocyte sedimentation rate (mm/h)	19 (11.25–34.00)	16 (11–34)	22 (14–35)	0.117
Fecal occult blood test (+)	70	38 (60.3)	32 (86.5)	0.006

### Drug treatment

The drugs used to treat the patients with mild IC are summarized in Table 3. Most of the patients were treated with probiotics (*n* = 68, 68%), followed by alanine glutamine (*n* = 56, 56%), and alprostadil injection (*n* = 46, 46%). Approximately 28% of the patients received empirical antibiotic therapy; however, this treatment was not associated with the hospital stay duration (*p* > 0.05). The proportion of patients who were hospitalized for ≤ 8 days and those who used probiotics was significantly greater than that of patients hospitalized for ≥ 12 days (76.2% vs. 54.1%, *p* = 0.022).

**Table 3:** Comparison of drug treatments and hospital stay duration for patients with mild IC

Drug treatment	Total	Hospital stay duration ≤ 8 days	Hospital stay duration ≥ 12 days	*p*-value
Patients	100	63	37	–
Alprostadil injection	46	29 (46.0)	17 (45.9)	0.99
Radix *Salvia miltiorrhiza* injection	20	11 (17.5)	9 (24.3)	0.41
Alanine glutamine	56	35 (55.6)	21 (56.8)	0.91
Probiotics	68	48 (76.2)	20 (54.1)	0.022
Antibiotics	28	17 (27.0)	11 (29.7)	0.77

### Predictors of in-hospital stay duration

In the logistic regression analysis, cerebrovascular disease history (*p* = 0.033), abdominal surgery history (*p* = 0.042), d-dimer level (*p* = 0.042), positive FOBT results (*p* = 0.003), and probiotic use (*p* = 0.011), were identified as significant factors associated with the hospital stay duration (Table 4); however, a history of coronary heart disease was not associated with the hospital stay duration (*p* > 0.05). Furthermore, patients with a history of cerebrovascular disease (OR = 4.585; 95% CI 1.129–18.624; *p* = 0.033) or abdominal surgery (OR = 4.551; 95% CI 1.060–19.546; *p* = 0.042) experienced a significantly longer hospital stay duration than did those without such histories. The hospital stay duration for patients with higher d-dimer levels or positive FOBT results was significantly longer than that of patients with lower d-dimer levels (OR = 1.928; 95% CI 1.024–3.632; *p* = 0.042) or those with negative FOBT results (OR = 7.211; 95% CI 1.929–26.953; *p* = 0.003). The patients who received probiotic treatment had a significantly shorter hospital stay duration than patients who did not receive probiotic treatment (OR = 0.237; 95% CI 0.078–0.717; *p* = 0.011).

**Table 4:** Factors associated with hospital stay duration

Variable	Hospital stay duration ≤ 8 days	Hospital stay duration ≥ 12 days	Univariable analysis			Multivariable analysis		
			OR	95% CI	*p*-value	OR	95% CI	*p*-value
History of cerebrovascular disease (vs. no)	7 (11.1)	11 (29.7)	3.385	1.178–9.727	0.024	4.585	1.129–18.624	0.033
History of coronary heart disease (vs. no)	9 (14.3)	10 (27.0)	2.222	0.808–6.114	0.122	1.503	0.412–5.482	0.537
History of abdominal surgery (vs. no)	5 (7.9)	11 (29.7)	4.908	1.548–15.560	0.007	4.551	1.060–19.546	0.042
d-dimer (mg/L)	0.28 (0.16–0.73)	0.78 (0.41–1.82)	2.021	1.175–3.477	0.011	1.928	1.024–3.632	0.042
Fecal occult blood test (positive vs. negative)	38 (60.3)	32 (86.5)	4.211	1.445–12.265	0.008	7.211	1.929–26.953	0.003
Use of probiotics (vs. no)	48 (76.2)	20 (54.1)	0.368	0.154–0.876	0.024	0.237	0.078–0.717	0.011

The multivariable analysis included histories of cerebrovascular disease, coronary heart disease, and abdominal surgery, d-dimer level, fecal occult blood test, and use of probiotics

*OR* odds ratio, *CI* confidence interval

## Discussion

To the best of our knowledge, this is the first study to evaluate the significant predictors that are associated with hospital stay duration for patients with mild IC; the results show several predictors of longer hospital stay duration for these patients. The multivariate model showed that patients with a history of cerebrovascular disease or abdominal surgery experienced a significantly longer hospital stay duration than did patients without such histories. Moreover, the hospital stay duration for patients with higher d-dimer levels or positive FOBT results was significantly longer than those of patients with lower d-dimer levels or negative FOBT results. Additionally, we observed that probiotic use contributed to a shortened hospital stay for the patients in this study. These findings highlight the importance of early identification of the risk factors that are associated with longer hospital stays, which is important in helping clinicians stratify patients based on overall disease severity and provide suitable therapeutic management.

The duration of symptoms in patients with IC is difficult to ascertain, considering the usual mild and benign course of the disease. The evolution of IC is significantly determined by the degree of ischemic damage to the colonic wall. Most patients show a good response to conservative treatment; their symptoms are expected to improve within 2–3 days, and complete clinical recovery is typically achieved within 2 weeks, with mucosal regeneration and healing occurring simultaneously [2, 10, 11]. A retrospective study reported that the hospital stay duration (mean value in days) was 13.53 days for alive patients with IC [12], which confirms that most patients with IC stay in the hospital for < 2 weeks. In our study cohort, the mean length of hospital stay was 9.6 days, while a hospital stay duration of ≥ 12 days for patients with mild IC is considered lengthy given the quick healing progress of mild lesions.

In our study, females accounted for 62 of the 100 patients, and the mean age of all patients was 61.50 ± 11.31 years. The results of this study reinforce the fact that the incidence of colonic ischemia is higher in female patients than in male patients, and IC is more common among older patients, as previously reported [8, 13]. This might be attributed to older patients’ increased susceptibility to atherosclerosis, which is one of the postmenopausal changes that leads to alterations in the blood supply of the colon, with a great impact on the areas of the colon most vulnerable to ischemic insult [14]. Previous studies indicated that abdominal pain, hematochezia, and diarrhea are the most common symptoms of IC, which is nearly consistent with the results of our study, as abdominal pain and rectal bleeding were the most frequent symptoms at presentation, followed by decreased appetite, abdominal distension, and diarrhea. Conversely, fever and weight loss were rare symptom presentations.

Prior studies have reported many risk factors that are predictive of mortality, such as advanced age, male sex, history of coronary artery disease and atrial fibrillation, peripheral vascular disease history, prior cardiovascular surgery, and dialysis dependence [15, 16]. A retrospective study conducted in Korea showed that 78% of the patients had comorbid conditions, with hypertension (46%) being the most common, followed by diabetes mellitus, nephropathy, and coronary artery disease. In that study, 48% of the patients had a history of surgical procedures, such as gastrointestinal tract surgery, abdominal aortic aneurysm surgery, or major cardiovascular surgery. Another study showed that the high mortality rate for patients with IC is due to associated comorbidities, such as ischemic heart disease, cerebrovascular disease, and peripheral vascular disease [9]. Further, our results were consistent with this finding, as the most common comorbidity in our study population was hypertension, followed by diabetes mellitus, hyperlipidemia, coronary heart disease, and cerebrovascular disease. Moreover, IC is usually a form of non-occlusive ischemia in the wall of the large intestine that is induced by a sudden reduction in the blood flow of the colonic vessels due to hypovolemia [17, 18]. Atherosclerotic disease, aortic surgery, and transient hypotension are assumed to be the causes in many cases; however, the etiology may be obscure for some patients. IC can also occur after cardiovascular surgery and surgical repair of abdominal aortic aneurysms, where it becomes an iatrogenic complication [19]. This could explain our finding that patients with a history of abdominal surgery had a significantly longer hospital stay length than those without such surgical history. Although the patients in this study had a higher incidence of coronary heart disease than cerebrovascular disease, our results indicate that coronary heart disease was not associated with the hospital stay duration. However, patients with a history of cerebrovascular disease had a significantly longer hospital stay duration than did the patients without a history of cerebrovascular disease.

An endoscopy is performed if the patient does not have peritonitis. Patients in the early stage of IC are frequently observed to have mucosal edema and fragility, segmental erythema, petechial hemorrhage, longitudinal ulcer, and lesions that are often segmented and patchy [20]. This validates our results, as erythematous colonic mucosa and edema were common colonoscopic findings in our patients, followed by multiple punctate erosions and superficial ulcers. No necrosis or deep ulcerations were detected in the patients.

Endoscopy results are usually related to a patient’s prognosis. For example, the left colon is commonly affected by lesions [21, 22]; however, involvement of the right colon is often related to higher mortality, need for surgery, and longer hospital stays [23, 24]. Our results show that right colonic involvement occurred more frequently in the patients who were hospitalized for ≥ 12 days than in those who were hospitalized for ≤ 8 days, although the difference was not significant (37.8% vs. 30.2%, *p* = 0.43).

Laboratory tests are often non-specific for IC, although reduced hemoglobin and bicarbonate concentrations or increased white blood cell or lactate dehydrogenase levels are often observed in patients with severe IC [8]. Although there is insufficient evidence to support the idea that these markers can help in the diagnosis of IC, measuring them in the early treatment process may provide a deeper insight into the IC severity of patients. Moreover, 28–72% of patients with IC had at least one type of thrombophilia, compared with 8.4% in the general population. Hypercoagulable states or secondary hyperfibrinolysis is also often observed in these patients with IC [25]. A previous study reported that hereditary and acquired thrombotic risk factors might play an important role in the pathogenesis of IC, and a tendency for thrombophilia was demonstrated in many patients with IC. The involvement of hereditary or acquired thrombotic risk factors is established in the pathogenesis of splanchnic vein thrombosis [26]. Thrombophilia and fibrinolysis can be confirmed based on d-dimer levels. In the present study, the d-dimer levels of patients with IC were higher than the normal range, consistent with the findings of a previous study [27]. Additionally, our results show that the hospital stay duration of patients with higher d-dimer levels was significantly longer than that of patients with lower d-dimer levels. In addition, a positive FOBT reflects the degree of ischemic damage in the colonic wall. We observed that the hospital stay duration for patients with positive FOBT results was significantly longer than for those with negative FOBT results.

The ACG clinical guidelines recommend that patients with mild diseases, such as transient IC, usually have a good prognosis and, therefore, do not require special treatment. However, for patients with remarkable symptoms or signs, hospitalization is recommended [8]. The major therapeutic measures currently used include intestinal rest, intravenous fluids, improvement of underlying conditions, and withdrawal of precipitated drugs [28]. In the previous decade, drugs used to treat IC were controversial; however, some promising new targets for medical therapy, such as prostaglandin E1, have been identified. Intravenous infusion of prostaglandin E1 leads to peripheral vessel vasodilation and increased colonic blood flow in animal models of IC, and it has been used for ischemic strictures in some case studies [29]. The results of our study indicate that 46 of the 100 patients with mild IC were treated with alprostadil injection; however, alprostadil use was not associated with a shorter hospital stay duration. In animal studies, antibiotic use was shown to shorten the disease duration, decrease the severity of IC, and prevent bacteria from translocating through the damaged mucosa [30, 31]. Although the ACG guidelines recommend that antibiotic treatment should be considered for moderate or severe IC [8], 28 of the 100 patients with mild IC in our study were treated with antibiotics, as prescribed by an attending physician. Our results indicate that antibiotic treatment may not significantly affect the hospital stay duration for patients with mild IC, which may be due to the inflammation caused by the use of antibiotics through the translocation of inherent colonic microflora. However, we found that probiotic use helped shorten the hospital stay for patients with mild IC, since the disease process can cause the destruction of the intestinal mucosal barrier, which may encourage bacteria to migrate into the circulatory system, whereas probiotic treatment can inhibit the infiltration and growth of pathogenic bacteria and maintain gut homeostasis [32]. Nevertheless, further studies, such as prospective randomized controlled trials, are warranted to confirm the role of probiotic therapies in the disease process.

This study has several limitations. First, it was a retrospective study; therefore, we could only report associations and could not establish causal relationships between predictors and length of hospital stay. Second, many clinical variables, such as lactate levels, serum procalcitonin levels, constipation rates, use of laxatives, and recent drug history, were unavailable. Third, the study period was more than 10 years, and it was subject to the inherent biases of retrospective analyses (i.e., detection bias). Finally, the number of patients diagnosed with IC was limited; thus, a larger sample size is necessary to conduct a large-scale study.

## Conclusions

In conclusion, patients with mild IC and cerebrovascular disease or an abdominal surgical history experienced significantly longer hospital stay duration than those without such medical histories. The length of hospital stay of patients with higher d-dimer levels or positive FOBT results was significantly longer than that of patients with lower d-dimer levels or negative FOBT results. However, probiotic use helped shorten the hospital stay duration for the patients. These findings emphasize the importance of early identification of risk factors associated with longer hospital stays and provide a clear target to develop better disease management strategies for patients with mild IC to shorten their hospital stay duration and consequently reduce the cost of healthcare.

## Data Availability

The datasets used and/or analyzed during the current study are available from the corresponding author on reasonable request.

## Abbreviations

IC: Ischemic colitis
FOBT: Fecal occult blood test
CT: Computed tomography
ACG: American College of Gastroenterology
ORs: Odds ratios
CIs: Confidence intervals
